# [Retracted] Antitumor activity of NRC-AN-019 in a pre-clinical breast cancer model

**DOI:** 10.3892/ijo.2026.5879

**Published:** 2026-03-30

**Authors:** Christopher S. Gondi, Bharathi Gorantla, A.K.S. Bhujanga Rao, K. Amala, M.U.R. Naidu, K.V. Jogi, G. Venkat Ramana, Praveen C. Myneni, Ajit Junnarkar, Jasti S. Rao

Int J Oncol 39: 641-648, 2011; DOI: 10.3892/ijo.2011.1079

Following the publication of the above paper, it was drawn to the Editor's attention by a concerned reader that certain of the 'Control' tumor images shown in Fig. 6A on p. 647 were unexpectedly similar to the tumor images shown five rows down for the 30 mg/kg Lapatinib administration experiment, albeit the images were turned through 180°.

Owing to the fact that this figure appeared to have been assembled erroneously, and that data may have been duplicated within the figure to show the results of apparently different experiments, the Editor of *International Journal of Oncology* has decided that this paper should be retracted from the Journal. The authors were asked for an explanation to account for these concerns, but the Editorial Office did not receive a reply. The Editor apologizes to the readership for any inconvenience caused.

